# Bis(tetra-*n*-butyl­ammonium) bis­(5,6-dicyano­pyrazine-2,3-dithiol­ato-κ^2^ *S*,*S*′)palladium(II)

**DOI:** 10.1107/S1600536811053402

**Published:** 2011-12-17

**Authors:** Masaaki Tomura, Yoshiro Yamashita

**Affiliations:** a Institute for Molecular Science, Myodaiji, Okazaki 444-8585, Japan; b Department of Electronic Chemistry, Interdisciplinary Graduate School of Science and Engineering, Tokyo Institute of Technology, Nagatsuta, Midori-ku, Yokohama 226-8502, Japan

## Abstract

In the title complex, (C_16_H_36_N)_2_[Pd(C_6_N_4_S_2_)_2_], the centrosymmetric dianion is planar, with an r.m.s. deviation of 0.034 (8) Å. The Pd^II^ atom, lying on an inversion center, has a square-planar coordination geometry, with Pd—S bond lengths of 2.276 (3) and 2.294 (3) Å.

## Related literature

For the synthesis of the title complex, see: Tomura *et al.* (1994 ▶). For mol­ecular conductors and superconductors based on metal dithiol­ene complexes, see: Brossard *et al.* (1986 ▶); Cassoux *et al.* (1991 ▶); Kobayashi *et al.* (1987 ▶); Tajima *et al.* (1993 ▶); Tanaka *et al.* (2001 ▶). For related structures, including the 2,3-dicyano-5,6-dimercaptopyrazine system, see: Belo *et al.* (1999 ▶, 2004 ▶); Nomura *et al.* (2009 ▶); Rabaça & Almeida (2010 ▶). For related structures, including the 1,2-dicyano-4,5-dimercaptobenzene system, see: Alves *et al.* (2004 ▶). For inter­molecular inter­actions caused by heteroatoms, see: Yamashita & Tomura (1998 ▶).
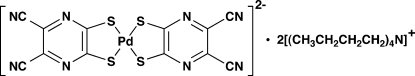


## Experimental

### 

#### Crystal data

(C_16_H_36_N)_2_[Pd(C_6_N_4_S_2_)_2_]
*M* *_r_* = 975.80
Triclinic, 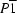

*a* = 9.912 (3) Å
*b* = 10.635 (4) Å
*c* = 13.286 (4) Å
α = 68.82 (2)°
β = 87.74 (3)°
γ = 80.16 (3)°
*V* = 1286.3 (8) Å^3^
*Z* = 1
Mo *K*α radiation
μ = 0.56 mm^−1^
*T* = 296 K
0.25 × 0.10 × 0.05 mm

#### Data collection

Rigaku AFC-7R diffractometer
5102 measured reflections
4835 independent reflections
1751 reflections with *I* > 2σ(*I*)
*R*_int_ = 0.087
3 standard reflections every 150 reflections  intensity decay: 1.0%

#### Refinement

*R*[*F* ^2^ > 2σ(*F* ^2^)] = 0.093
*wR*(*F* ^2^) = 0.222
*S* = 0.98
4835 reflections
272 parameters
H-atom parameters constrained
Δρ_max_ = 0.39 e Å^−3^
Δρ_min_ = −0.41 e Å^−3^

### 

Data collection: *MSC/AFC Diffractometer Control Software* (Molecular Structure Corporation, 1988 ▶); cell refinement: *MSC/AFC Diffractometer Control Software*; data reduction: *TEXSAN* (Molecular Structure Corporation & Rigaku, 2000 ▶); program(s) used to solve structure: *SIR97* (Altomare *et al.*, 1999 ▶); program(s) used to refine structure: *SHELXL97* (Sheldrick, 2008 ▶); molecular graphics: *PLATON* (Spek, 2009 ▶); software used to prepare material for publication: *SHELXL97*.

## Supplementary Material

Crystal structure: contains datablock(s) global, I. DOI: 10.1107/S1600536811053402/hy2494sup1.cif

Structure factors: contains datablock(s) I. DOI: 10.1107/S1600536811053402/hy2494Isup2.hkl

Additional supplementary materials:  crystallographic information; 3D view; checkCIF report

## Figures and Tables

**Fig. 1.**
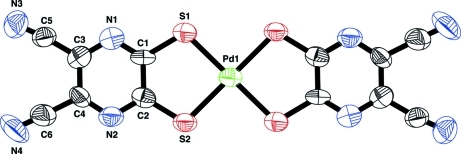
The molecular structure of the dianion in (I). Displacement ellipsoids are drawn at the 50% probability level. Unlabeled atoms are related to the labeled atoms by -x+2, -y-1, -z.

**Fig. 2.**
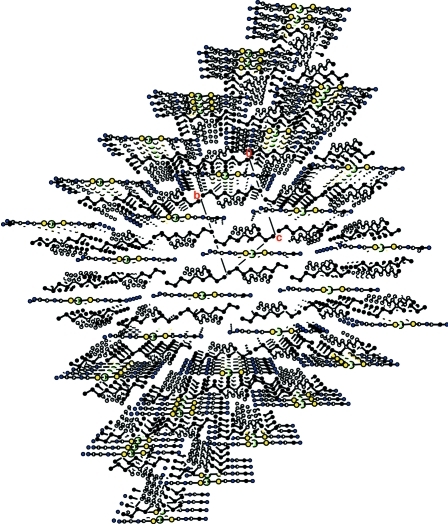
The perspective view of the crystal packing of (I), viewed along the a axis. H atoms have been omitted for clarity.

## supplementary crystallographic information

### Comment

Metal dithiolene complexes have been widely investigated as molecular conductors and superconductors. Several superconductors (Brossard *et al.*, 1986; Cassoux *et al.*, 1991; Kobayashi *et al.*, 1987; Tajima *et al.*, 1993) and single-component molecular metals (Tanaka *et al.*, 2001) involving dithiolene complexes have been discovered to date. We have synthesized the title palladium dithiolene complex, (I), derived from 2,3-dicyano-5,6-dimercaptopyrazine ligand (Tomura *et al.*, 1994). The ligand is expected to extend the π-conjugation of the complex resulting in decreased Coulombic repulsion (Belo *et al.*, 1999, 2004; Nomura *et al.*, 2009; Rabaça & Almeida, 2010). Intermolecular interactions caused by S···S and S···N heteroatom contacts may increase the dimensionality in the solid state (Yamashita & Tomura, 1998). We report here the molecular and crystal structures of (I).

In the complex (I)  the dianion molecule is located on an inversion center. The molecular structure of the dianion is shown in Fig. 1. The dianion is a flat molecule with an r.m.s. deviation of 0.034 (8) Å of fitted atoms from the least-squares plane. The central Pd atom has a square-planar coordination geometry and the Pd1—S1 and Pd1—S2 distances and the S1—Pd1—S2 angle are 2.276 (3), 2.294 (3) Å and 89.39 (10)°, respectively. These values are comparable to those found in bis(tetra-n-butylammonium) bis(4,5-dicyanobenzene-1,2-dithiolato-S,S')palladium(II) complex (Alves *et al.*, 2004). Fig. 2 shows the packing diagram of (I) viewed along the *a* axis. The dianion molecules form a layered structure with an interlayer distance of 6.5 Å. The tetra-n-butylammonium cations are inserted between the layers.

### Experimental

The title compound (I) was synthesized according to the literature method (Tomura *et al.*, 1994). Orange crystals of (I) suitable for X-ray analysis were grown from an acetone solution.

### Refinement

All H atoms were placed in geometrically calculated positions and refined using a riding model, with C—H = 0.97 (methylene) and 0.96 (methyl) Å and *U*_iso_(H) = 1.2(1.5 for methyl)*U*_eq_(C).

### Crystal data

**Table:**

(C_16_H_36_N)_2_[Pd(C_6_N_4_S_2_)_2_]	*Z* = 1
*M**_r_* = 975.80	*F*(000) = 516
Triclinic, *P*1	*D*_x_ = 1.260 Mg m^−^^3^
Hall symbol: -P 1	Melting point: 520.5 K
*a* = 9.912 (3) Å	Mo *K*α radiation, λ = 0.7107 Å
*b* = 10.635 (4) Å	Cell parameters from 24 reflections
*c* = 13.286 (4) Å	θ = 20.1–22.7°
α = 68.82 (2)°	µ = 0.56 mm^−^^1^
β = 87.74 (3)°	*T* = 296 K
γ = 80.16 (3)°	Needle, orange
*V* = 1286.3 (8) Å^3^	0.25 × 0.10 × 0.05 mm

### Data collection

**Table:**

Rigaku AFC-7R diffractometer	*R*_int_ = 0.087
Radiation source: rotating anode	θ_max_ = 27.5°, θ_min_ = 1.6°
graphite monochromator	*h* = −12→11
ω–2θ scans	*k* = −12→0
5102 measured reflections	*l* = −15→14
4835 independent reflections	3 standard reflections every 150 reflections
1751 reflections with *I* > 2σ(*I*)	intensity decay: 1.0%

### Refinement

**Table:**

Refinement on *F*^2^	Primary atom site location: structure-invariant direct methods
Least-squares matrix: full	Secondary atom site location: difference Fourier map
*R*[*F*^2^ > 2σ(*F*^2^)] = 0.093	Hydrogen site location: inferred from neighbouring sites
*wR*(*F*^2^) = 0.222	H-atom parameters constrained
*S* = 0.98	*w* = 1/[σ^2^(*F*_o_^2^) + (0.0782*P*)^2^] where *P* = (*F*_o_^2^ + 2*F*_c_^2^)/3
4835 reflections	(Δ/σ)_max_ < 0.001
272 parameters	Δρ_max_ = 0.39 e Å^−^^3^
0 restraints	Δρ_min_ = −0.41 e Å^−^^3^

### Special details

**Table:**

Geometry. All e.s.d.'s (except the e.s.d. in the dihedral angle between two l.s. planes) are estimated using the full covariance matrix. The cell e.s.d.'s are taken into account individually in the estimation of e.s.d.'s in distances, angles and torsion angles; correlations between e.s.d.'s in cell parameters are only used when they are defined by crystal symmetry. An approximate (isotropic) treatment of cell e.s.d.'s is used for estimating e.s.d.'s involving l.s. planes.
Refinement. Refinement of *F*^2^ against ALL reflections. The weighted *R*-factor *wR* and goodness of fit *S* are based on *F*^2^, conventional *R*-factors *R* are based on *F*, with *F* set to zero for negative *F*^2^. The threshold expression of *F*^2^ > σ(*F*^2^) is used only for calculating *R*-factors(gt) *etc*. and is not relevant to the choice of reflections for refinement. *R*-factors based on *F*^2^ are statistically about twice as large as those based on *F*, and *R*- factors based on ALL data will be even larger.

### Fractional atomic coordinates and isotropic or equivalent isotropic displacement parameters (Å^2^)

**Table:**

	*x*	*y*	*z*	*U*_iso_*/*U*_eq_	
Pd1	1.0000	0.5000	1.0000	0.0590 (5)	
S1	1.0651 (3)	0.7047 (3)	0.9048 (2)	0.0750 (9)	
S2	0.7779 (3)	0.5945 (3)	0.9425 (2)	0.0677 (9)	
N1	0.9253 (10)	0.9342 (10)	0.7768 (7)	0.063 (2)	
N2	0.6744 (9)	0.8402 (11)	0.8066 (8)	0.073 (3)	
N3	0.8122 (11)	1.2596 (11)	0.5958 (9)	0.095 (3)	
N4	0.4777 (11)	1.1342 (12)	0.6318 (11)	0.127 (5)	
N5	0.4587 (7)	0.5033 (8)	0.7369 (6)	0.052 (2)	
C1	0.9162 (9)	0.8100 (12)	0.8408 (7)	0.045 (3)	
C2	0.7894 (11)	0.7582 (11)	0.8586 (8)	0.060 (3)	
C3	0.8115 (12)	1.0150 (12)	0.7253 (9)	0.064 (3)	
C4	0.6864 (11)	0.9648 (13)	0.7422 (10)	0.071 (3)	
C5	0.8184 (11)	1.1533 (14)	0.6519 (10)	0.069 (3)	
C6	0.5674 (13)	1.0600 (13)	0.6803 (10)	0.083 (4)	
C7	0.5687 (9)	0.5275 (10)	0.6517 (7)	0.063 (3)	
H7A	0.6080	0.4405	0.6458	0.075*	
H7B	0.5256	0.5859	0.5825	0.075*	
C8	0.6833 (10)	0.5921 (11)	0.6738 (8)	0.073 (3)	
H8A	0.7260	0.5351	0.7435	0.087*	
H8B	0.6452	0.6807	0.6774	0.087*	
C9	0.7918 (11)	0.6105 (11)	0.5879 (8)	0.075 (3)	
H9A	0.7503	0.6704	0.5185	0.090*	
H9B	0.8284	0.5226	0.5826	0.090*	
C10	0.9092 (11)	0.6720 (12)	0.6152 (9)	0.095 (4)	
H10A	0.8725	0.7576	0.6229	0.142*	
H10B	0.9739	0.6868	0.5581	0.142*	
H10C	0.9541	0.6100	0.6815	0.142*	
C11	0.3911 (9)	0.6377 (9)	0.7481 (7)	0.054 (3)	
H11A	0.3212	0.6181	0.8021	0.065*	
H11B	0.4597	0.6734	0.7751	0.065*	
C12	0.3266 (12)	0.7474 (11)	0.6475 (9)	0.084 (3)	
H12A	0.2521	0.7167	0.6225	0.100*	
H12B	0.3938	0.7660	0.5912	0.100*	
C13	0.2724 (13)	0.8766 (12)	0.6698 (10)	0.100 (4)	
H13A	0.2081	0.8558	0.7281	0.120*	
H13B	0.3480	0.9069	0.6936	0.120*	
C14	0.2024 (14)	0.9909 (12)	0.5733 (11)	0.129 (5)	
H14A	0.2686	1.0215	0.5191	0.193*	
H14B	0.1604	1.0655	0.5946	0.193*	
H14C	0.1335	0.9589	0.5447	0.193*	
C15	0.3575 (9)	0.4356 (10)	0.7003 (7)	0.063 (3)	
H15A	0.4077	0.3556	0.6886	0.076*	
H15B	0.3173	0.4985	0.6314	0.076*	
C16	0.2417 (10)	0.3915 (11)	0.7775 (8)	0.071 (3)	
H16A	0.1865	0.4712	0.7863	0.085*	
H16B	0.2801	0.3307	0.8477	0.085*	
C17	0.1509 (10)	0.3179 (11)	0.7340 (8)	0.075 (3)	
H17A	0.1097	0.3800	0.6652	0.090*	
H17B	0.2070	0.2405	0.7223	0.090*	
C18	0.0388 (11)	0.2682 (13)	0.8123 (10)	0.109 (5)	
H18A	0.0793	0.2112	0.8818	0.163*	
H18B	−0.0117	0.2167	0.7859	0.163*	
H18C	−0.0218	0.3455	0.8188	0.163*	
C19	0.5197 (10)	0.4106 (11)	0.8485 (8)	0.063 (3)	
H19A	0.5845	0.4567	0.8687	0.076*	
H19B	0.4464	0.4019	0.8998	0.076*	
C20	0.5915 (11)	0.2686 (11)	0.8611 (8)	0.075 (3)	
H20A	0.6722	0.2735	0.8171	0.090*	
H20B	0.5309	0.2209	0.8382	0.090*	
C21	0.6318 (13)	0.1934 (12)	0.9792 (9)	0.099 (4)	
H21A	0.6889	0.2447	1.0014	0.119*	
H21B	0.5498	0.1893	1.0217	0.119*	
C22	0.7070 (16)	0.0519 (14)	1.0024 (11)	0.144 (6)	
H22A	0.6491	−0.0012	0.9846	0.216*	
H22B	0.7324	0.0107	1.0777	0.216*	
H22C	0.7878	0.0549	0.9600	0.216*	

### Atomic displacement parameters (Å^2^)

**Table:**

	*U*^11^	*U*^22^	*U*^33^	*U*^12^	*U*^13^	*U*^23^
Pd1	0.0540 (9)	0.0755 (11)	0.0521 (8)	−0.0223 (7)	0.0036 (6)	−0.0235 (7)
S1	0.0555 (19)	0.082 (2)	0.081 (2)	−0.0214 (17)	0.0010 (16)	−0.0172 (18)
S2	0.0515 (18)	0.084 (2)	0.0647 (19)	−0.0243 (16)	0.0047 (14)	−0.0173 (17)
N1	0.073 (7)	0.072 (7)	0.059 (6)	−0.019 (6)	0.009 (5)	−0.037 (6)
N2	0.055 (6)	0.071 (7)	0.098 (7)	−0.017 (6)	0.014 (5)	−0.035 (6)
N3	0.108 (9)	0.065 (8)	0.111 (9)	−0.014 (7)	−0.009 (7)	−0.031 (7)
N4	0.071 (8)	0.124 (11)	0.190 (13)	0.012 (7)	−0.055 (8)	−0.069 (9)
N5	0.048 (5)	0.061 (6)	0.051 (5)	−0.009 (4)	0.002 (4)	−0.027 (5)
C1	0.041 (6)	0.078 (8)	0.038 (6)	−0.028 (6)	0.015 (5)	−0.040 (6)
C2	0.050 (7)	0.072 (8)	0.071 (7)	−0.008 (6)	0.009 (6)	−0.042 (7)
C3	0.069 (8)	0.059 (9)	0.074 (8)	−0.011 (7)	0.024 (7)	−0.036 (7)
C4	0.043 (7)	0.063 (9)	0.102 (10)	0.003 (7)	−0.007 (6)	−0.029 (8)
C5	0.062 (8)	0.068 (10)	0.081 (9)	−0.022 (7)	0.006 (6)	−0.028 (8)
C6	0.070 (9)	0.083 (10)	0.103 (10)	−0.022 (8)	−0.006 (8)	−0.038 (8)
C7	0.055 (7)	0.068 (8)	0.055 (6)	−0.003 (6)	0.002 (5)	−0.014 (6)
C8	0.057 (7)	0.084 (9)	0.071 (7)	−0.017 (6)	0.008 (6)	−0.019 (6)
C9	0.081 (8)	0.062 (8)	0.075 (8)	−0.007 (7)	0.017 (6)	−0.019 (6)
C10	0.071 (8)	0.102 (10)	0.097 (9)	−0.031 (8)	0.013 (7)	−0.014 (8)
C11	0.054 (6)	0.048 (7)	0.056 (6)	0.010 (5)	0.007 (5)	−0.023 (6)
C12	0.078 (8)	0.065 (8)	0.101 (10)	0.003 (7)	−0.004 (7)	−0.029 (8)
C13	0.101 (10)	0.072 (10)	0.119 (11)	−0.018 (8)	0.029 (9)	−0.026 (9)
C14	0.143 (13)	0.075 (10)	0.134 (12)	0.028 (9)	−0.015 (10)	−0.016 (10)
C15	0.063 (7)	0.062 (7)	0.064 (7)	−0.015 (6)	−0.012 (6)	−0.019 (6)
C16	0.059 (7)	0.089 (9)	0.068 (7)	−0.011 (7)	0.006 (6)	−0.035 (7)
C17	0.064 (7)	0.087 (9)	0.082 (8)	−0.014 (7)	−0.010 (6)	−0.039 (7)
C18	0.088 (9)	0.138 (12)	0.129 (11)	−0.062 (9)	0.021 (8)	−0.066 (10)
C19	0.056 (7)	0.076 (9)	0.072 (8)	−0.026 (6)	0.007 (6)	−0.038 (7)
C20	0.079 (8)	0.080 (9)	0.072 (8)	−0.013 (7)	−0.005 (6)	−0.033 (7)
C21	0.102 (10)	0.084 (10)	0.094 (10)	0.000 (8)	−0.038 (8)	−0.014 (8)
C22	0.174 (16)	0.094 (12)	0.128 (12)	0.010 (11)	−0.030 (11)	−0.008 (10)

### Geometric parameters (Å, °)

**Table:**

Pd1—S1^i^	2.276 (3)	C11—H11B	0.9700
Pd1—S1	2.276 (3)	C12—C13	1.514 (14)
Pd1—S2	2.294 (3)	C12—H12A	0.9700
Pd1—S2^i^	2.294 (3)	C12—H12B	0.9700
S1—C1	1.732 (10)	C13—C14	1.503 (15)
S2—C2	1.716 (11)	C13—H13A	0.9700
N1—C1	1.304 (11)	C13—H13B	0.9700
N1—C3	1.336 (12)	C14—H14A	0.9600
N2—C4	1.315 (12)	C14—H14B	0.9600
N2—C2	1.351 (12)	C14—H14C	0.9600
N3—C5	1.100 (12)	C15—C16	1.527 (12)
N4—C6	1.126 (13)	C15—H15A	0.9700
N5—C15	1.516 (10)	C15—H15B	0.9700
N5—C7	1.525 (10)	C16—C17	1.537 (12)
N5—C11	1.527 (10)	C16—H16A	0.9700
N5—C19	1.532 (11)	C16—H16B	0.9700
C1—C2	1.434 (12)	C17—C18	1.519 (13)
C3—C4	1.412 (14)	C17—H17A	0.9700
C3—C5	1.452 (15)	C17—H17B	0.9700
C4—C6	1.469 (16)	C18—H18A	0.9600
C7—C8	1.513 (12)	C18—H18B	0.9600
C7—H7A	0.9700	C18—H18C	0.9600
C7—H7B	0.9700	C19—C20	1.508 (13)
C8—C9	1.520 (12)	C19—H19A	0.9700
C8—H8A	0.9700	C19—H19B	0.9700
C8—H8B	0.9700	C20—C21	1.514 (13)
C9—C10	1.542 (13)	C20—H20A	0.9700
C9—H9A	0.9700	C20—H20B	0.9700
C9—H9B	0.9700	C21—C22	1.488 (15)
C10—H10A	0.9600	C21—H21A	0.9700
C10—H10B	0.9600	C21—H21B	0.9700
C10—H10C	0.9600	C22—H22A	0.9600
C11—C12	1.497 (12)	C22—H22B	0.9600
C11—H11A	0.9700	C22—H22C	0.9600
			
S1^i^—Pd1—S1	180.00 (15)	C11—C12—H12B	109.7
S1^i^—Pd1—S2	90.61 (10)	C13—C12—H12B	109.7
S1—Pd1—S2	89.39 (10)	H12A—C12—H12B	108.2
S1^i^—Pd1—S2^i^	89.39 (10)	C14—C13—C12	113.4 (11)
S1—Pd1—S2^i^	90.61 (10)	C14—C13—H13A	108.9
S2—Pd1—S2^i^	180.0	C12—C13—H13A	108.9
C1—S1—Pd1	104.8 (4)	C14—C13—H13B	108.9
C2—S2—Pd1	103.6 (4)	C12—C13—H13B	108.9
C1—N1—C3	118.1 (9)	H13A—C13—H13B	107.7
C4—N2—C2	117.0 (9)	C13—C14—H14A	109.5
C15—N5—C7	105.9 (7)	C13—C14—H14B	109.5
C15—N5—C11	112.2 (7)	H14A—C14—H14B	109.5
C7—N5—C11	110.8 (7)	C13—C14—H14C	109.5
C15—N5—C19	110.3 (7)	H14A—C14—H14C	109.5
C7—N5—C19	111.4 (7)	H14B—C14—H14C	109.5
C11—N5—C19	106.3 (7)	N5—C15—C16	115.8 (7)
N1—C1—C2	122.7 (11)	N5—C15—H15A	108.3
N1—C1—S1	117.7 (7)	C16—C15—H15A	108.3
C2—C1—S1	119.6 (9)	N5—C15—H15B	108.3
N2—C2—C1	119.2 (11)	C16—C15—H15B	108.3
N2—C2—S2	118.3 (8)	H15A—C15—H15B	107.4
C1—C2—S2	122.5 (9)	C15—C16—C17	110.7 (8)
N1—C3—C4	119.6 (11)	C15—C16—H16A	109.5
N1—C3—C5	119.5 (10)	C17—C16—H16A	109.5
C4—C3—C5	120.8 (12)	C15—C16—H16B	109.5
N2—C4—C3	123.5 (11)	C17—C16—H16B	109.5
N2—C4—C6	120.8 (10)	H16A—C16—H16B	108.1
C3—C4—C6	115.7 (12)	C18—C17—C16	111.2 (8)
N3—C5—C3	174.2 (13)	C18—C17—H17A	109.4
N4—C6—C4	178.9 (15)	C16—C17—H17A	109.4
C8—C7—N5	114.8 (8)	C18—C17—H17B	109.4
C8—C7—H7A	108.6	C16—C17—H17B	109.4
N5—C7—H7A	108.6	H17A—C17—H17B	108.0
C8—C7—H7B	108.6	C17—C18—H18A	109.5
N5—C7—H7B	108.6	C17—C18—H18B	109.5
H7A—C7—H7B	107.5	H18A—C18—H18B	109.5
C7—C8—C9	112.6 (9)	C17—C18—H18C	109.5
C7—C8—H8A	109.1	H18A—C18—H18C	109.5
C9—C8—H8A	109.1	H18B—C18—H18C	109.5
C7—C8—H8B	109.1	C20—C19—N5	117.6 (8)
C9—C8—H8B	109.1	C20—C19—H19A	107.9
H8A—C8—H8B	107.8	N5—C19—H19A	107.9
C8—C9—C10	111.1 (9)	C20—C19—H19B	107.9
C8—C9—H9A	109.4	N5—C19—H19B	107.9
C10—C9—H9A	109.4	H19A—C19—H19B	107.2
C8—C9—H9B	109.4	C19—C20—C21	107.8 (9)
C10—C9—H9B	109.4	C19—C20—H20A	110.1
H9A—C9—H9B	108.0	C21—C20—H20A	110.1
C9—C10—H10A	109.5	C19—C20—H20B	110.1
C9—C10—H10B	109.5	C21—C20—H20B	110.1
H10A—C10—H10B	109.5	H20A—C20—H20B	108.5
C9—C10—H10C	109.5	C22—C21—C20	113.3 (11)
H10A—C10—H10C	109.5	C22—C21—H21A	108.9
H10B—C10—H10C	109.5	C20—C21—H21A	108.9
C12—C11—N5	116.3 (8)	C22—C21—H21B	108.9
C12—C11—H11A	108.2	C20—C21—H21B	108.9
N5—C11—H11A	108.2	H21A—C21—H21B	107.7
C12—C11—H11B	108.2	C21—C22—H22A	109.5
N5—C11—H11B	108.2	C21—C22—H22B	109.5
H11A—C11—H11B	107.4	H22A—C22—H22B	109.5
C11—C12—C13	110.0 (9)	C21—C22—H22C	109.5
C11—C12—H12A	109.7	H22A—C22—H22C	109.5
C13—C12—H12A	109.7	H22B—C22—H22C	109.5
			
S2—Pd1—S1—C1	−2.2 (3)	N1—C3—C4—C6	−178.7 (9)
S2^i^—Pd1—S1—C1	177.8 (3)	C5—C3—C4—C6	0.8 (15)
S1^i^—Pd1—S2—C2	−177.6 (3)	C15—N5—C7—C8	−178.6 (9)
S1—Pd1—S2—C2	2.4 (3)	C11—N5—C7—C8	59.4 (10)
C3—N1—C1—C2	−0.7 (13)	C19—N5—C7—C8	−58.7 (11)
C3—N1—C1—S1	178.5 (7)	N5—C7—C8—C9	178.5 (8)
Pd1—S1—C1—N1	−177.7 (6)	C7—C8—C9—C10	−178.1 (9)
Pd1—S1—C1—C2	1.6 (7)	C15—N5—C11—C12	−60.9 (10)
C4—N2—C2—C1	−0.4 (14)	C7—N5—C11—C12	57.3 (10)
C4—N2—C2—S2	−179.5 (8)	C19—N5—C11—C12	178.5 (8)
N1—C1—C2—N2	0.6 (14)	N5—C11—C12—C13	−176.3 (8)
S1—C1—C2—N2	−178.6 (7)	C11—C12—C13—C14	−178.4 (10)
N1—C1—C2—S2	179.7 (7)	C7—N5—C15—C16	175.8 (8)
S1—C1—C2—S2	0.5 (11)	C11—N5—C15—C16	−63.1 (11)
Pd1—S2—C2—N2	176.8 (7)	C19—N5—C15—C16	55.2 (11)
Pd1—S2—C2—C1	−2.3 (8)	N5—C15—C16—C17	−177.1 (8)
C1—N1—C3—C4	0.6 (14)	C15—C16—C17—C18	177.6 (10)
C1—N1—C3—C5	−178.9 (8)	C15—N5—C19—C20	57.1 (10)
C2—N2—C4—C3	0.3 (16)	C7—N5—C19—C20	−60.2 (11)
C2—N2—C4—C6	178.5 (9)	C11—N5—C19—C20	179.0 (8)
N1—C3—C4—N2	−0.4 (16)	N5—C19—C20—C21	−175.2 (8)
C5—C3—C4—N2	179.2 (10)	C19—C20—C21—C22	−178.6 (11)

Symmetry codes: (i) −*x*+2, −*y*+1, −*z*+2.
